# From a conventional maneuver to a mechanical repositioning procedure for torsional-vertical down beating positioning nystagmus based on numerical simulations

**DOI:** 10.1007/s00405-026-10329-2

**Published:** 2026-06-01

**Authors:** Ismael Arán-Tapia, Nicolás Pérez-Fernández, Manuel Sánchez-Yáñez, Octavio Garaycochea

**Affiliations:** 1 Group of Non-Linear Physics, University of Santiago de Compostela, Campus Sur, Santiago de Compostela, Spain; 2 Cross-Disciplinary Research Center in Environmental Technologies (CRETUS), University of Santiago de Compostela, Santiago de Compostela, Spain; 3 Department of Otorhinolaryngology, Clínica Universidad de Navarra, Madrid, 28027 Spain; 4 Galician Center for Mathematical Research and Technology (CITMAga), Santiago de Compostela, Spain; 5 Department of Otorhinolaryngology, Clínica Universidad de Navarra, Avenida Pio XII 36, 31008 Pamplona, Spain

**Keywords:** Benign paroxysmal positional vertigo, Down beating nystagmus, Maneuver, Mechanical repositioning chair, Computational fluid dynamics, Mathematical modeling

## Abstract

**Purpose:**

To explore, using numerical simulations, the biomechanical behavior of otoconia during the Garaycochea maneuver in torsional-vertical down-beating positioning nystagmus (TVP-DBNy) associated with benign paroxysmal positional vertigo (BPPV), and to propose a modified maneuver designed for execution under controlled conditions compatible with a mechanical-repositioning-chair (MRC).

**Methods:**

A high-resolution three-dimensional micro–computed tomography (μCT) reconstruction of a human membranous labyrinth was used to simulate TVP-DBNyrelated conditions. The main clinical scenarios producing TVP-DBNy where included: short-arm of the posterior canal (PC); distal portion of the long-arm of the PC; and a contralateral anterior canal (AC). Endolymphatic flow was modeled using the Navier–Stokes equations, and otoconia of different sizes (5–20 μm) were introduced as Lagrangian particles. Two maneuver protocols were simulated: the original Garaycochea maneuver and a modified sequence simulated under idealized, controlled conditions consistent with MRC-based execution.

**Results:**

The original maneuver did not consistently promote otoconial transport toward the utricular macula across all simulated scenarios. The modified maneuver resulted in trajectories that favored otoconial progression toward the utricle under the specific kinematic conditions simulated, with limited displacement observed for the smallest particles.

**Conclusion:**

The simulations suggest that symptom improvement after the standard maneuver may be associated with scenarios in which most particles remain near the utricular side of the horizontal ampulla. The proposed modification provides mechanistic insight into possible personalized repositioning strategies but should be interpreted as exploratory and hypothesis-generating, conditional on the modeled conditions, rather than as evidence of clinical superiority.

## Introduction

Benign paroxysmal positional vertigo (BPPV) is currently understood to result from the abnormal presence of otoconia from the utricular macula (UM) in the semicircular canals (SCC). Changes in head position within the plane of the affected canal generate abnormal endolymphatic flow, leading to cupular deflection and modulation of vestibular afferent activity. This results in recurrent episodes of positional nystagmus associated with vertigo, whose duration correlates with that of the nystagmus. Therapeutic positional maneuvers are highly effective in the treatment of BPPV, as they promote the migration of otoconia out of the canal and back into the utricle, where they are presumed to be reabsorbed or dissolved [1].

The posterior canal (PC) is the most frequently affected, most commonly due to canalithiasis of the proximal or periampullary segment of the long arm. Conventional maneuvers, such as Sémont’s liberatory maneuver and the Epley canalith repositioning procedure, are widely used to treat typical PC-BPPV by facilitating otoconial movement through inertial forces or gravity. Despite their overall effectiveness, treatment failures still occur, particularly in non-typical forms of PC-BPPV [2].

The PC can also be affected in locations other than the typical site. Atypical posterior canal BPPV (PCv-BPPV), including the canalithiasis of the distal portion of the long arm or the short arm, may produce a torsional-vertical down-beating positioning nystagmus (TVP-DBNy) during head-hanging positions or the Dix-Hallpike test, mimicking anterior canal (AC) BPPV [3, 4]. Differentiating between these entities can be clinically challenging, and in some cases, the diagnosis becomes evident only after conversion to a typical form following treatment [5].

A maneuver addressing both anterior canal BPPV on one side and contralateral PCv-BPPV has been described (Garaycochea maneuver) [6] and may be useful in cases of TVP-DBNy, where the underlying mechanism cannot be reliably distinguished clinically. This approach aims to convert atypical PC involvement into a typical form while simultaneously resolving AC involvement. However, a considerable proportion of patients require repeated maneuvers, suggesting limitations in its effectiveness that are not yet fully understood. Recently, mechanical repositioning chairs (MRCs) have been introduced as a therapeutic option for refractory cases of BPPV [7]. These devices allow controlled 360-degree rotation and precise positioning, reducing inaccuracies associated with manual maneuvers. They also enable treatment to be adapted to individual anatomical variations of the SCC, which may contribute to maneuver failure [7].

Therefore, the aim of this study is to investigate possible biomechanical factors contributing to the failure of the Garaycochea maneuver. Using computational fluid dynamics (CFD) simulations, we analyze endolymphatic flow and otoconial motion within a membranous labyrinth reconstructed from high-resolution micro–computed tomography (µCT) data during the maneuver. In addition, this study explores the feasibility of deriving an alternative, subject-specific repositioning maneuver based on the use of mechanical repositioning chairs (MRCs).

## Materials and methods

### Mathematical model

The anatomical model consists of a left membranous labyrinth reconstructed from high-resolution µCT data [8], with the contralateral labyrinth obtained by mirror symmetry. Consequently, the model represents a single subject-specific anatomy and does not capture true inter-ear or inter-individual anatomical variability. The reconstructed geometry was refined to facilitate mathematical analysis and meshing. A mesh independence study yielded a polyhedral grid of 351,716 cells per labyrinth, representing the endolymphatic fluid region [9]. Simulations were performed with a time-step of 1 ms.

The membranous labyrinth walls, including the cupula, were modeled as rigid with a no-slip condition, since elasticity is negligible at the frequencies relevant for repositioning procedures [10, 11], and neither cupular nor wall deformations are expected to generate flows or displacements capable of significantly altering otoconia motion.

**Fig. 1:**
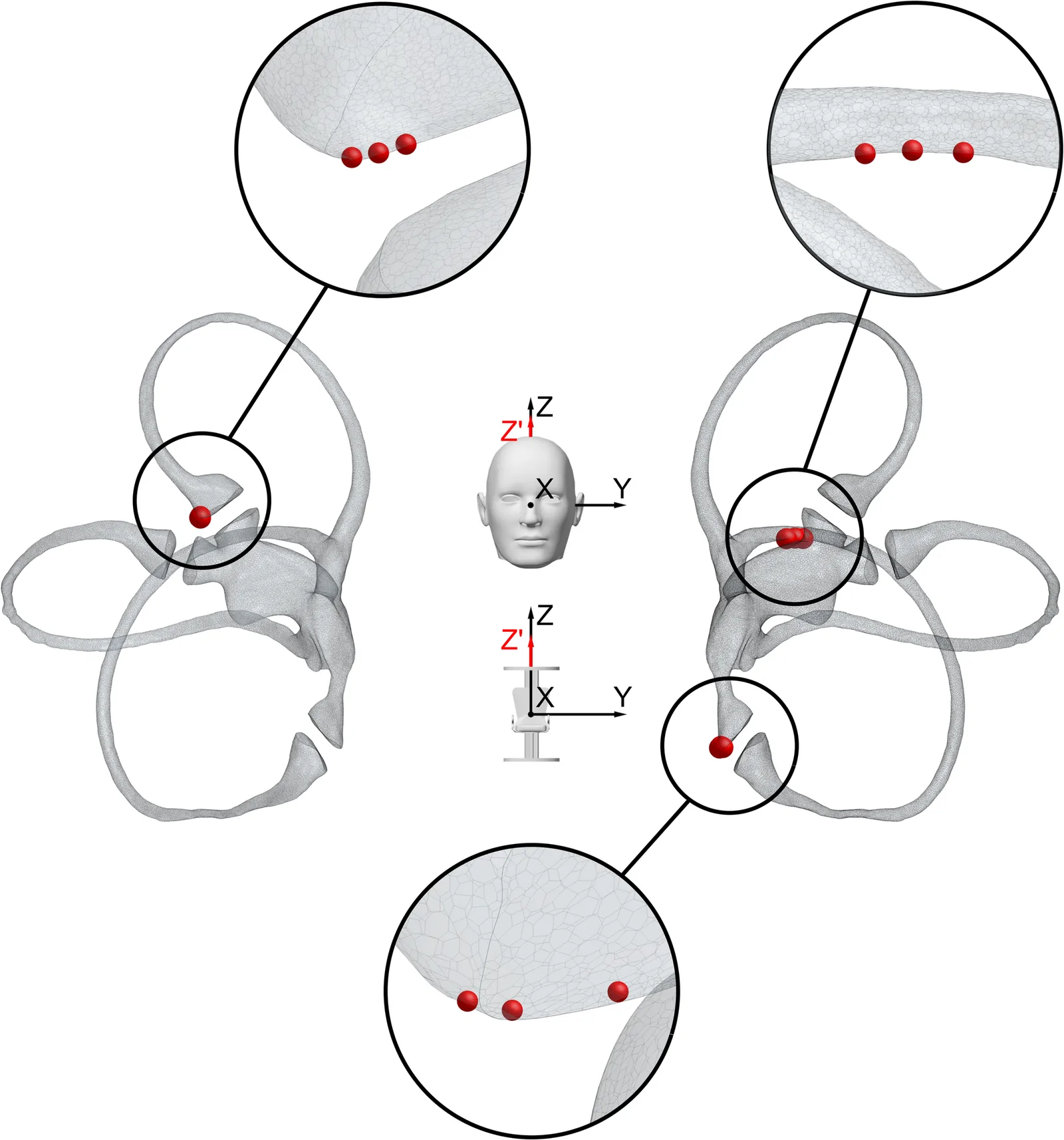
Anatomical meshes of the left and right membranous labyrinths illustrating the initial positions of the otoconia. Three distinct regions were selected, with three input points in each, highlighted in the zoomed views: right anterior proximal portion of the long arm, left posterior short arm, and left posterior distal portion of the long arm. The central panel shows the initial orientation of the head and waist coordinate systems for the standard maneuver, and of the long- and short-arm coordinate systems for the personalized maneuver in the rotational chair

Otoconia were placed at several initial positions to mimic the main clinical scenarios producing similar nystagmus patterns: (i) left-PC, short arm; (ii) left-PC, distal portion of the long (non-ampullary) arm; and (iii) right-AC, proximal portion of the long (ampullary) arm. For each region, three initial positions around the equilibrium posture in supine were considered (see Fig. 1). At each position, one otoconium of each representative size (20 μm, 15 μm, 10 μm, and 5 μm) was introduced, yielding three particles per size in each region.

The simulations were performed using the computational fluid dynamics (CFD) software Simcenter STAR-CCM+. Otoconia were modeled as Lagrangian particles and endolymph flow with the Navier–Stokes equations, both models including inertial, gravitational, drag, and buoyancy forces [9]. Because of the low particle-to-fluid volume ratio [12], particle–particle collisions were neglected to reduce computational cost. The endolymph was modeled as a laminar fluid with a dynamic viscosity of 8.15·10⁻⁴ Pa·s and a density of 1000 kg/m³ [8], whereas the otoconia were assigned a density of 2700 kg/m³ [13].

### Standard maneuver

The standard definition of the maneuver was simulated following the procedure originally described [6], including the prescribed rotational angles and timing of each step. In this definition, certain rotations were performed with respect to either the head or the waist origin (see Table 1), which were separated by 75 cm [14]. Positive rotations indicate counterclockwise motion with respect to a given axis, whereas negative values indicate clockwise motion.

**Table 1:** Definition of steps and substeps in the standard maneuver, specifying the rotation origin, axis, and angle. Angular convention: + denotes counterclockwise, – denotes clockwise

Step	Axis of rotation	Origin	Rotation (º)
1 A	Y’	Head	45
1B	Z’	Head	45
2	Y	Waist	90
3	Z’	Head	-90
4	Y	Waist	-90
5 A	Z’	Head	45
5B	Y’	Head	-45

The maneuver was defined as being performed on a bed. Under this condition, the head coordinate system moves whenever rotations are executed with respect to the waist coordinate system, replicating the natural behavior of the head rotating relative to the waist. Initially, both coordinate systems were aligned, as illustrated in Fig. 1, where the Z axis of the waist coordinate system coincides with the Z′ axis of the head coordinate system. For clarity, the waist system was kept fixed in the diagrams, which facilitates comparison across steps. This convention is geometrically consistent, as all waist rotations occur about the Y axis.

### Personalized maneuver

The personalized maneuver was designed exclusively for use with MRCs, as certain positions required for success cannot be achieved on a bed. In this case, all rotations are performed with respect to the patient’s waist origin. MRCs, however, allow motion only about two axes: rotations around the long-arm X axis and rotations around the short-arm Z′ axis (see Fig. 1). Under this consideration, the coordinate system of the long arm remains fixed—equivalent to keeping the waist system fixed in the standard maneuver—while the short-arm coordinate system rotates whenever rotations around the long-arm X axis are performed. Based on the same criteria as in Tables 1 and 2 summarizes the sequence of positions defined for each step of the maneuver, including the final orientations achieved along the two available axes. These cumulative angles correspond solely to the imposed rotational sequence of the simulated maneuver and are included as a descriptive kinematic reference. They do not represent a physiological measure or the simulated otoconial displacement.

**Table 2:** Definition of steps and substeps in the personalized maneuver, specifying the rotation origin, axis, and angle. The rotation angles were selected based on the numerical simulation results. Cumulative angles represent the algebraic sum of the chair rotations and are reported as a kinematic descriptor of the maneuver sequence illustrated in Fig. 3. Angular convention: + denotes counterclockwise, – denotes clockwise

Step	Axis of rotation	Origin	Rotation (º)	Cumulative short-arm (º)	Cumulative long-arm (º)
1 A	Z’	Waist	120	120	0
1B	X	Waist	-80	120	-80
2 A	Z’	Waist	40	160	-80
2B	X	Waist	-70	160	-150
3 A	Z’	Waist	10	170	-150
3B	X	Waist	-110	170	-260
4 A	Z’	Waist	-20	150	-260
4B	X	Waist	-60	150	-320
5 A	Z’	Waist	-60	90	-320
5B	X	Waist	-110	90	-430

Each rotation in the simulation lasted 3 s, followed by a 60-second resting period. The selected rotation duration, angular velocity profile, and resting times were chosen as representative values reported in clinical practice and were intended to define a controlled reference condition rather than an optimized or exhaustive exploration of maneuver dynamics. Accordingly, no systematic parameter sweeps or sensitivity analyses were performed with respect to angular velocity or acceleration.

Because otoconia do not move continuously whiting the semicircular canals, the sequence of rotations could not be derived analytically. Instead, multiple simulations were performed in which the rotation angle was varied in 10° increments to identify steps that promoted otoconial progression within the constraints of the selected protocol. The final sequence was selected based on a qualitative assessment of otoconial behavior in both membranous labyrinths. This maneuver was therefore derived empirically through iterative simulation rather than through formal mathematical optimization. The resulting sequence is not necessarily unique, and alternative angular combinations or timing conditions could potentially produce similar or improved outcomes. Accordingly, the observed results should be interpreted as conditional on the specific kinematic parameters defined in the present simulations.

## Results

### Standard maneuver

**Fig. 2:**
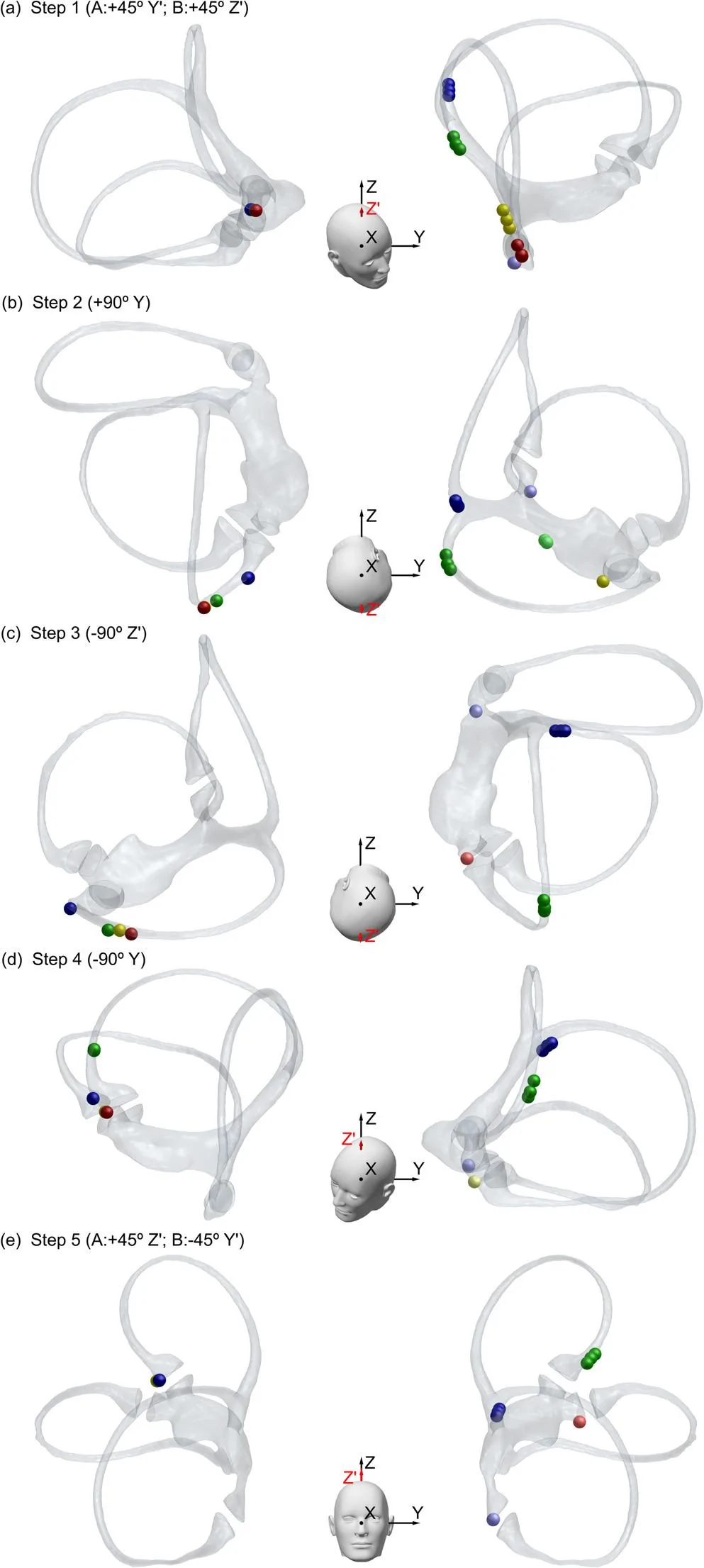
Head and membranous labyrinth orientations during each step of the standard maneuver for the simultaneous treatment of left- and right-sided otoconia. Otoconia are depicted as enlarged spheres with the following color code: 20 μm (red), 15 μm (yellow), 10 μm (green), and 5 μm (blue). Particles originating from the posterior short arm are shown in desaturated colors to distinguish them from those starting in the posterior distal portion of the long arm. The figure illustrates the final otoconia positions after the resting period of (**a**) Step 1, (**b**) Step 2, (**c**) Step 3, (**d**) Step 4, and (**e**) Step 5

The results of the standard maneuver simulation are shown in Fig. 2, with the detailed sequence of rotations provided in Table 1.

Figure 2a shows the position of the otoconia after 30s of resting following a head rotation. In this step, otoconia from the left posterior distal portion of the long arm migrate towards the posterior short arm. Particles larger than 15-µm cross the common crus (CC) and move closer to the posterior ampulla, whereas otoconia of 10-µm or smaller do not progress sufficiently and remain at the entrance of the CC. On the right side, otoconia move slightly away from the anterior cupula.

Figure 2b shows the final position after 60s of resting following a waist rotation. In this step, most left sided otoconia reach the utricle, except for the 5-µm particles, which show minimal translation, and the 10-µm otoconia originating in the left posterior distal portion of the long arm. The latter, which had previously migrated towards the CC, now enter the left anterior canal. On the right side, otoconia exit the anterior ampulla, advancing slightly along the right anterior canal.

Figure 2c shows the final position after 60s of resting following another head rotation. This orientation favors further progression of the 10-µm otoconia that entered the left-AC in the previous step. The smaller otoconia remain in the same location, while those that had already reached the utricle are now grouped near the utricular side of the left anterior ampulla. On the right side, otoconia show little displacement due to the lateral orientation of the right-AC.

Figure 2d shows the final position after the patient is returned to a seated posture while maintaining the head orientation for 30s. This step only advances the 10-µm otoconia deeper into the left-SC. Otoconia previously grouped in the utricular side of the anterior ampulla now move towards the corresponding side of the horizontal ampulla. On the right side, larger otoconia return to the anterior ampulla, while the 10-µm particles do not have sufficient time to move back during the resting period.

Figure 2e shows the final position after the patient is placed upright and remains there for 30s. In this step, left-sided otoconia are dispersed in positions different from those observed earlier in the maneuver, but none reach the UM. Most particles remain in the utricular side of the horizontal ampulla, while the 10-µm otoconia originating from the left posterior distal long arm move closer to the anterior ampulla after traversing the entire AC. The 5-µm otoconia remain nearly unchanged from their initial position, although over a longer period they are expected to accumulate in the short arm. On the right side, otoconia remain at their initial location in the proximal portion of the right anterior long arm, independent of particle size.

### Personalized maneuver

**Fig. 3:**
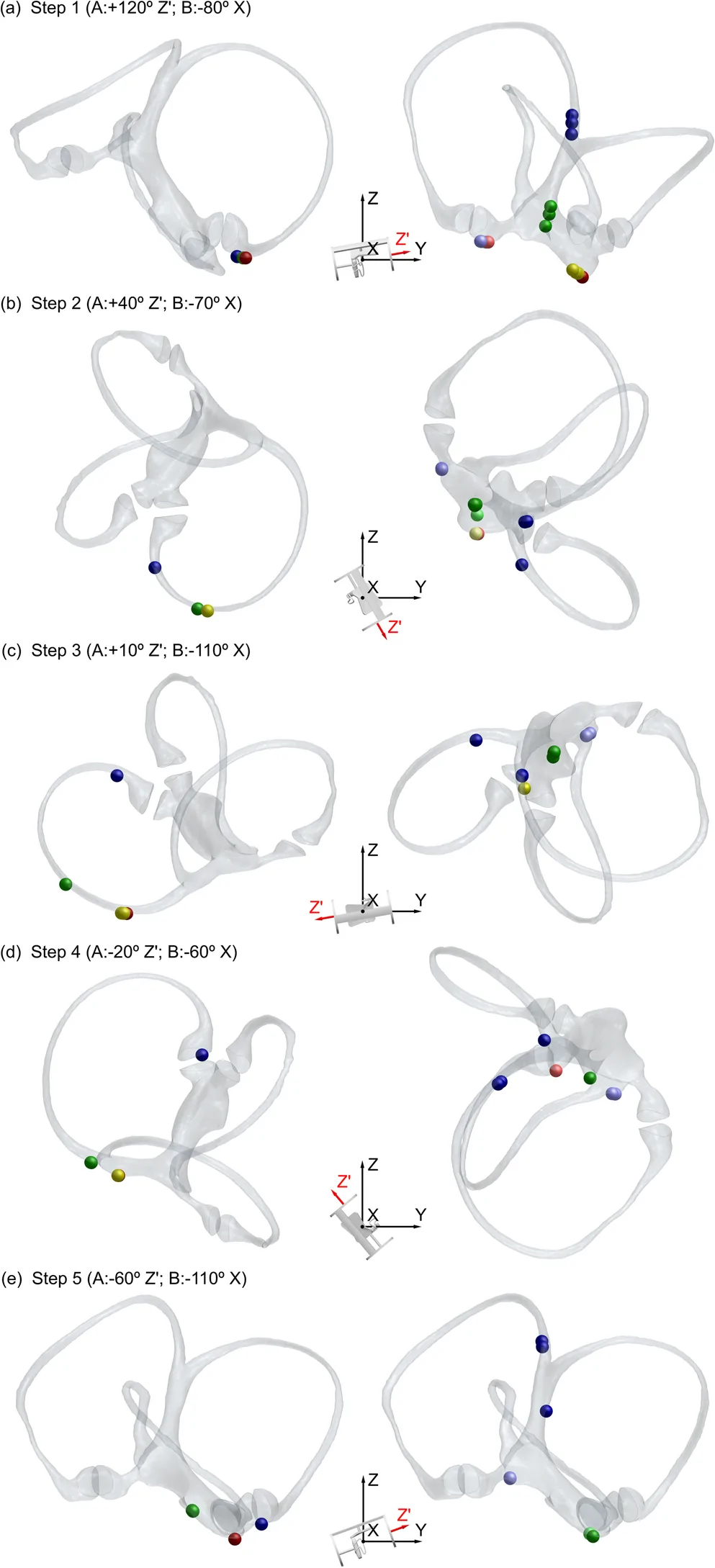
Rotary chair and membranous labyrinth orientations during each step of the personalized maneuver for simultaneous treatment of left- and right-sided otoconia. Each step included a 60 s resting period. Otoconia are depicted as enlarged spheres with the following color code: 20 μm (red), 15 μm (yellow), 10 μm (green), and 5 μm (blue). Particles originating from the posterior short arm are shown in desaturated colors to distinguish them from those starting in the posterior distal portion of the long arm. The figure illustrates the final otoconia positions after the resting period of (**a**) Step 1, (**b**) Step 2, (**c**) Step 3, (**d**) Step 4, and (**e**) Step 5

The results of the personalized maneuver simulation are shown in Fig. 3 for each step after 60s of resting time, with the detailed sequence of rotations provided in Table 2.

Figure 3a shows the final position during step 1. In this step, otoconia originating from the left posterior distal portion of the long arm migrate towards the utricle, crossing the CC except for the 5-µm particles. Otoconia starting in the left posterior short arm and in the right anterior proximal portion of the long arm remain approximately in the same location, moving only slightly away from their respective cupula.

Figure 3b corresponds to Step 2. In this position, otoconia from the left posterior short arm advance through the utricle, joining those that migrated in the previous step. The 5-µm particles exit the posterior ampulla, and some otoconia from the long arm enter the AC. On the right side, most otoconia advance significantly along the canal, except for the 5-µm particles.

Figure 3c shows Step 3, which allows the right-sided otoconia to continue their progression along the canal, although the 5-µm particles remain trapped in the proximal portion of the right anterior long arm. On the left side, otoconia previously located in the utricle remain there, while the 5-µm particles from the left posterior distal long arm now begin to move away from the CC.

Figure 3d shows Step 4. In this position, right sided otoconia reach the CC, except for the 5-µm particles, which remain in the right anterior ampulla. On the left side, most otoconia stay in the utricle, with some located in the utricular side of the horizontal ampulla.

Figure 3e shows the final step. Here, otoconia from the right side reach the UM for all particle sizes, except for the 5 μm otoconia, which return to their initial location in the proximal portion of the right anterior long arm. On the left side, otoconia of all sizes reach the UM except for the 5 μm particles, which are expected to eventually arrive at the same position given an indefinite resting period.

## Discussion

Physical therapy maneuvers are considered the gold standard first-line treatment of BPPV. Clinical resolution in typical variants is afforded by one or more maneuvers in 70 to 95% of cases [15–17], however, in less common subtypes or variants, the therapeutic response may be reduced or absent, and recurrence very short [18]. In this simulated clinical scenario of TVP-DBNy-BPPV, the standard Garaycochea maneuver did not achieve consistent otoconial transport to the UM in the AC-BPPV condition, nor did it reliably convert contralateral PCv-BPPV into a typical posterior canal form. From this numerical simulation, the limited effectiveness of the maneuver may be associated with AC-BPPV cases in which otoconia remain near their initial location, or with contralateral PCv-BPPV cases in which 10 μm otoconia from the long arm migrate into the AC, as well as with the accumulation of smaller (5 μm) particles in the short arm. Conversely, improvement after the standard maneuver may be consistent with scenarios in which most particles remain in the utricular side of the horizontal ampulla, adjacent to the UM [8, 19].

In contrast, the personalized maneuver resulted in simulated trajectories that favored otoconial transport toward the UM within the simulation, under the specific kinematic conditions considered for most particle sizes and configurations. For the smallest otoconia (5 μm), the simulations showed minimal translational motion, with particles remaining close to their initial positions throughout successive steps of the maneuver. This behavior is consistent with the expected slow settling dynamics of very small particles [9, 13]. Under these conditions, longer resting times or alternative motion profiles would likely be required to further mobilize such particles, although the exploration of these scenarios is beyond the scope of the present study. Regarding maneuver design, an initial objective was to explore a simultaneous bilateral repositioning strategy to reduce the number of steps and the overall maneuver duration. However, within the modeling framework used, no satisfactory solution was identified. Consequently, a sequential approach was adopted, first addressing the left side and subsequently the right, while attempting to maintain previously repositioned otoconia within the utricle. This outcome highlights the complexity of designing bilateral repositioning maneuvers and supports the need for subject-specific approaches, particularly in atypical forms of BPPV. Importantly, this study does not evaluate clinical efficacy or demonstrate therapeutic superiority, but rather illustrates how controlled kinematic execution within a computational framework may influence simulated otoconial transport under the modeled conditions.

In patients with TVP-DBNy-BPPV, the torsional component may help identify the affected side (right versus left), whether due to AC-BPPV or PCv-BPPV; however, distinguishing between these two scenarios remains clinically difficult. In AC-BPPV, this may be related to the proximity of the AC to the sagittal plane (41° compared to the 56° for PC), whereas in PCv-BPPV it may be associated to the inhibitory nature of the stimulus [6]. In some cases, the definitive diagnosis becomes evident only after conversion to a typical form following treatment [5, 6, 18]. At present, therapeutic alternatives for TVP-DBNy-BPPV are limited. The Yacovino maneuver is one of the most commonly used for AC-BPPV [20], but it assumes anterior canal involvement and is applied symmetrically, regardless of the affected side. Reported success rates are highly variable, ranging from 36% to 100% [5, 21]. This variability may reflect geometric mismatch between the maneuver plane and the affected canal, as well as interindividual differences in semicircular canal orientation [22]. In addition, some cases may correspond to PCv-BPPV rather than true AC-BPPV, which could further contribute to treatment failure. For PCv-BPPV cases in which the affected side is known, maneuvers such as the Demi-Semont or the Quick liberatory maneuver have been proposed [3, 18]. However, these approaches remain limited by diagnostic uncertainty and anatomical variability, which have motivated the development of modified maneuvers based on Unity 3D/Maya animations and numerical CFD micro-CT [23, 24].

According to the International Classification of Vestibular Disorders, vestibular disorders are primarily defined and classified based on clinical signs and symptoms, while the underlying pathophysiological mechanisms may remain incompletely established [25]. In this context, biomechanical and computational models should be considered as complementary tools to explore possible mechanisms and generate hypotheses, rather than as substitutes for clinical validation or standardized treatment recommendations. Several biomechanical and computational studies have explored SCC dynamics and otoconial behavior under controlled conditions, providing mechanistic insight into BPPV without constituting clinical validation. Vestibular responses are known to depend on velocity- and acceleration-related dynamics, and different stimulus intensities may produce different responses, even in normal subjects [26, 27]. Numerical models have shown that endolymph flow and otoconial trajectories are sensitive to canal geometry, particle size, and head orientation, and that small variations in these parameters may lead to different outcomes [13, 28]. More recent CFD-based studies using three-dimensional reconstructions have supported the usefulness of these approaches for explaining maneuver variability and failure, while also emphasizing their limitations and the need for cautious interpretation [9, 24, 29]. Therefore, the results of the present study should be interpreted as mechanistic and hypothesis-generating rather than as confirmatory of clinical effectiveness.

MRCs allow precise control of rotational angles in yaw, roll, and pitch axes, facilitating the execution of complex movements that may be difficult to achieve manually. Other potential advantages compared to conventional maneuvers (CMs) include improved analytical feasibility, accurate navigation, treatment of patients unfit for manual maneuvers, and reduced physical effort for the clinician [30]. In the context of the present study, the use of MRCs should be interpreted as an experimental tool that enables the controlled execution of the personalized maneuver under idealized conditions, rather than as evidence of clinical superiority over CMs. Although previous studies have suggested potential benefits of MRCs in selected or refractory cases [30–32], clinical effectiveness, robustness under real-world variability, and patient tolerance were not evaluated in this work. In this study, MRCs primarily serve as a platform to illustrate how complex, subject-specific motion sequences derived from numerical simulations can be implemented under controlled conditions.

An important limitation of this study is that the membranous labyrinth model was reconstructed from a single human specimen, and the contralateral labyrinth was generated by mirror-image reconstruction, which limits anatomical variability and precludes generalization to other individuals. This approach inherently limits anatomical variability and constrains bilateral interpretation, since interindividual and inter-ear differences in semicircular canal orientation are well documented. Previous CT-based have reported deviations from theoretical orthogonality of up to 7–8° and discrepancies as large as 20° relative to cranial landmarks [22]. Therefore, the present findings are strictly anatomy-specific, and any bilateral observations should be interpreted as illustrative of possible fluid-dynamic behavior rather than as representative of general anatomical conditions. In addition, head-motion inputs, including rotation duration, angular velocity profile, and resting times, were not systematically varied. Therefore, the present results should be interpreted as a subject-specific, mechanistic proof of concept focused on fluid-dynamic behavior under the specific kinematic conditions simulated, rather than as a universally generalizable or population-level anatomical or dynamic representation. Future studies incorporating multiple, independently reconstructed patient-specific anatomies and a broader exploration of maneuver dynamics may help determine whether subject-specific modeling can provide insight into alternative repositioning strategies.

In summary, the present simulations suggest that the standard Garaycochea maneuver may explain symptom improvement in those cases in which otoconia remain on the utricular side of the horizontal ampulla after the maneuver, close to the utricular macula, even if complete repositioning is not achieved. At the same time, the model shows that not all particles reach the utricle, which may help explain why clinical improvement is not consistently observed. These findings are conditional on the specific kinematic conditions simulated, and their robustness to variations in acceleration profile, rotation velocity, or resting time was not assessed. By integrating realistic canal geometry, otoconial size, and controlled motion sequences within a physics-based framework, the simulations provide mechanistic insight into why atypical BPPV presentations may respond differently to standard maneuvers and why complete repositioning with a single maneuver may be difficult to achieve under the modeled conditions. In this context, carefully designed, physically consistent numerical simulations may represent a valuable tool to generate hypotheses and to guide future studies aimed at developing more individualized repositioning strategies.

## Data Availability

All data generated or analyzed during the present study has been incorporated into this published article. The original datasets used and analyzed during the current study are available from the corresponding author upon reasonable request.
